# Recent Advances on In Situ SEM Mechanical and Electrical Characterization of Low-Dimensional Nanomaterials

**DOI:** 10.1155/2017/1985149

**Published:** 2017-10-25

**Authors:** Chenchen Jiang, Haojian Lu, Hongti Zhang, Yajing Shen, Yang Lu

**Affiliations:** ^1^Department of Mechanical and Biomedical Engineering, City University of Hong Kong, Kowloon, Hong Kong; ^2^Centre for Advanced Structural Materials (CASM), Shenzhen Research Institute, City University of Hong Kong, Shenzhen 518057, China; ^3^Centre for Robotics and Automation (CRA), Shenzhen Research Institute, City University of Hong Kong, Shenzhen 518057, China

## Abstract

In the past decades, in situ scanning electron microscopy (SEM) has become a powerful technique for the experimental study of low-dimensional (1D/2D) nanomaterials, since it can provide unprecedented details for individual nanostructures upon mechanical and electrical stimulus and thus uncover the fundamental deformation and failure mechanisms for their device applications. In this overview, we summarized recent developments on in situ SEM-based mechanical and electrical characterization techniques including tensile, compression, bending, and electrical property probing on individual nanostructures, as well as the state-of-the-art electromechanical coupling analysis. In addition, the advantages and disadvantages of in situ SEM tests were also discussed with some possible solutions to address the challenges. Furthermore, critical challenges were also discussed for the development and design of robust in situ SEM characterization platform with higher resolution and wider range of samples. These experimental efforts have offered in-depth understanding on the mechanical and electrical properties of low-dimensional nanomaterial components and given guidelines for their further structural and functional applications.

## 1. Introduction

Due to their excellent mechanical and electrical properties, low-dimensional (1D/2D) nanomaterials, such as metallic/polymer/semiconductor nanowires, graphene, and MoS_2_, have become important building blocks in applications like nanoelectronics, solar cells, and sensors, and so on [1–4]. Therefore, it is necessary to get a thorough understanding of their mechanical behaviors and electrical properties for the purposes of exploring their full potential functions and promoting the development of the advanced micro/nanoelectronics applications and mechatronic systems. However, due to their exceedingly small sample sizes at micro- and nanoscales, people can merely observe their general morphologies under optical microscopes [5] before, while they cannot directly manipulate and characterize them until the recent breakthroughs in scanning electron microscopy (SEM).

With the recent development of scanning electron microscopy and small scale micro/nanomanipulation and mechanical/electrical testing techniques, interrogating the unique and wide-spectrum properties of individual nanostructures directly inside scanning electron microscopes (SEM) became possible. Various kinds of characterization methods for different types of nanomaterials, such as tensile tests, compression tests, and bending tests, have come forth. Particularly, as to the metallic or semiconductor nanomaterials, investigating their electrical properties is also meaningful. Traditionally, these tests can only be done outside SEM given to the limited chamber size of the testing instruments and controlling mechanisms. Although people can acquire data such as strength and Young's modulus of these nanomaterials and derive their failure mechanism by performing postmortem SEM study, they lose the opportunity to know how the samples behave upon mechanical/electrical stimulus which may contain abundant interesting phenomena. Therefore, people have spent years of efforts on developing small testing platforms which were suitable for in situ SEM mechanical or electrical characterizations. In recent years, with the help of commercialized nanoindentation system, atomic force microscope (AFM), micro/nanofabrication method, and micromechanical and microelectromechanical system (MEMS) devices, these testing methods can be gradually combined together for the desired “in situ characterization” inside SEM.

On the other hand, in order to give researchers more freedom and higher precision ability to manipulate the nanomaterials inside SEM during in situ experiments, advanced robotic systems have also been developed. Besides the function in nanomaterial sample manipulation and transferring, these systems also can exert force and electricity stimuli on samples directly during tests. Therefore, in this review paper, we mainly focus on the latest development on the in situ SEM techniques for individual nanomaterial testing including the recent micro/nanorobotics advances in this application field, as well as the related nanomaterial manipulation and transferring techniques with the corresponding challenges discussed.

## 2. In Situ SEM Mechanical Tests of Micro/Nanomaterials

### 2.1. Tensile Testing of 1D or 2D Nanostructures

Among all the mechanical testing techniques, tensile test is the most straightforward manner which can provide a wide-spectrum of mechanical properties, such as elasticity, plasticity, and fracture strength, in a direct way. Since in situ SEM tensile test has been developed by Dingley [6], large amount of efforts has been devoted to this field [7–9]. The size effects of nanomaterials have been demonstrated by in situ tensile mechanical test for many materials, such as Ag nanowire [10] and ZnO nanowire [11]. In most of the cases, the fracture strength increases as the diameter of the nanowires decreases. Recently, a new concept of “ultra-strength” has been proposed [12, 13] and further demonstrated in many nanomaterials. Tian et al. [14] approached the elastic strain limit of the submicron-sized metallic glass specimens and the corresponding strength of them was about twice as high as the already impressive elastic limit observed in bulk metallic glass samples. Zhang et al. [15] have found that vapor-liquid-solid–grown single-crystalline Si nanowires with diameters of ~100 nm could be repeatedly stretched above 10% elastic strain at room temperature, approaching the theoretical elastic limit of silicon (17 to 20%). However, not every nanomaterial will display such “smaller is stronger” size effect; Zhang et al. [16] conducted in situ uniaxial quasi-static tensile tests on individual nanocrystalline Co nanowires and observed that Young's modulus is (75.3 ± 14.6) GPa with a tensile strength of (1.6 ± 0.4) GPa, which are significantly lower than their bulk counterparts and the theoretical value of monocrystalline samples, therefore, deviated from the traditional theory.

**Figure 1:**
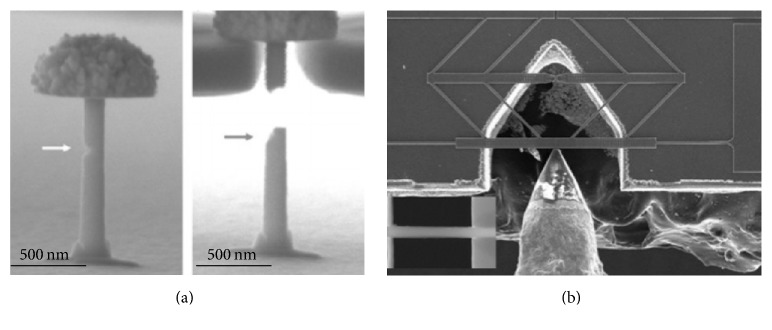
Typical tensile testing configurations on nanostructures. (a) shows that the sample can be stretched by a custom-milled diamond tension grip in SEM [17]. (b) Push-to-pull micromechanical device which can convert the compression force of the nanoindenter into tensile force [18].

Understanding the failure mechanism in micro/nanomaterials is demanding for the design of reliable structural materials and micro- and nanoscale devices. Gu et al. [17] investigated the fracture behavior of nanocrystalline Pt nanocylinders with prefabricated surface notches as shown in Figure 1(a) and demonstrated that most of these samples fractured at the notches. Fatigue fracture mechanism of nanomaterials also can be done with in situ tensile loading; Lu et al. [18, 19] have demonstrated the first quantitative low-cycle in situ SEM tensile fatigue testing of Ni nanowires based on the nanoindenter-assisted “push-to-pull MEMS” dynamic tensile straining system, as shown in Figure 1(b). Also based on MEMS device, Jiang et al. [20] developed a high cycle nanowire fatigue tensile and torsion platform which reduced the time to investigate the fatigue behavior of nanostructures.

In situ SEM tensile test can also fulfill the mechanical investigation of 2D nanostructures. Therefore, metal thin films, which are key components in microelectronics devices, have been studied extensively. Haque and Saif [24] presented a novel tensile testing technique utilizing MEMS force sensors for in situ mechanical characterization of submicron scale freestanding thin films in SEM decade ago. Sim and Vlassak [25] studied the mechanical properties of thin Au films at various temperature and strain rates during in situ SEM tensile tests. An inverse size effect where the yield strength at elevated temperature decreases with decreasing temperature was also observed. Zhang et al. [26] reported the first in situ tensile testing of suspended graphene using a nanomechanical device in a SEM and found that the cracked graphene samples exhibit a fast brittle fracture behavior with the breaking stress much lower than the intrinsic strength of graphene. Recently, we also investigated the 2D MoS_2_ membranes under in situ SEM tensile loading and provided some critical insights into the mechanical properties and fracture behavior of them [27].

### 2.2. Compression Testing of Micro/Nanopillars

**Figure 2:**
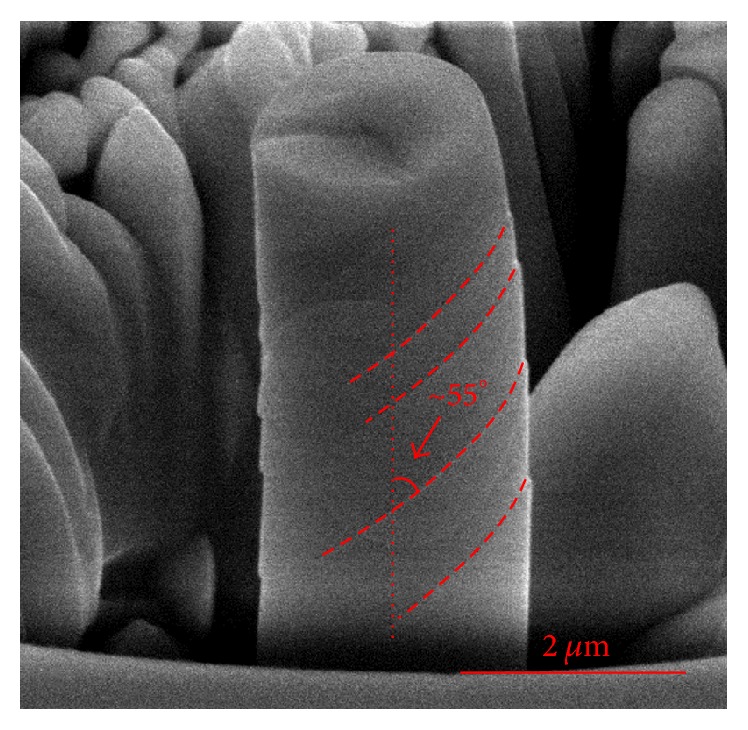
The typical postcompression test of niobium nanopillar which fractured with pronounced slip offsets [21].

Compression tests on micro/nanomaterials are similar to that applied on the macroscopic samples, but with some modifications facilitating both the fabrication of the diminutive samples and the subsequent manipulation via the testing system. Commercial nanoindentation systems are always regarded as the mechanical test frame of compression experiments, except that the sharp indentation tip is accordingly replaced with a flat-punch tip. The load and displacement resolutions of most nanoindentation systems are well suited for micro/nanocompression testing because they typically produce stress-strain curves with nanoscale resolution for micro/nanoscale samples.  Figure 2 shows the typical compression test of a nanopillar which was fabricated by FIB.

As many micro/nanomaterials behave significantly different in compression tests from the way they perform under tension [28], the compression testing of micromachined micro/nanopillars is currently an active research area since Gane and Bowden [29] firstly reported the in situ compression test inside SEM. The failure mechanism of materials under compression may be the most attracting point to the researchers because of the particular stress state in it, which is usually not entirely uniaxial. This approach has sparked a number of studies and the traditional laws of plasticity at small scales were challenged because the overall sample dimensions limited the length scales available for plastic processes [30–33]. Particularly, for amorphous MG (metallic glass) materials, which usually have high strength, low inhomogeneous plasticity of micro/nanopillars have been found under compression tests [34].

Similar to the in situ tensile test of micro/nanomaterials, size effects were also observed in compression tests in both bcc and fcc single-crystalline micro/nanopillars [35]. Kim and Greer [36] even conducted a contrastive in situ tensile and compression tests on fcc (Au) and bcc (Mo) nanopillars and found that the size dependence between the two loading directions in Au nanopillar was identical while there was a pronounced tension-compression asymmetry in Mo nanopillars.

Recently, compression tests at different circumstances or on special materials were also conducted. Wheeler and Michler [37] investigated the transitions in deformation mechanism of silicon nanopillars with increasing temperature under microcompression test. Raghavan et al. [38] studied the failure mechanism of Cu/TiN multilayered thin film micropillars at elevated temperature and found that the yielding of the multilayers was governed by the stress-assisted diffusion of the Cu interlayers, which coalesce into microcrystals and grow into larger faceted crystals at elevated temperatures of 200 and 400°C. Zhang et al. [21] systematically investigated the CoCrCuFeNi high-entropy alloy micro/nanopillars, which has equi- or near equiatomic compositions and found the less sensitive size effect of its yield strength. Traditionally, the semiconductor materials are usually brittle at room temperature; however, Michler et al. [39] found that the GaAs micropillars have very large plastic strain even comparable to that of metal single crystal micropillars.

### 2.3. Nanoindentation on Thin Films

Nanoindentation system is not only suitable for micro/nanopillars compression test, but also useful in the quantitative characterization of thin films [40] and microbeams with custom made tips. As some ceramic thin films are widely used as a protective coating in tribological applications [41], it became necessary to investigate the microhardness, Young's modulus, and fracture toughness of them. With the decreasing size of the actuators and sensors, the in situ SEM nanoindentation can give more information on the formation and propagation of mechanically induced dislocations and defects during the experiment so as to correlate the load-displacement data with the in situ microstructural changes. For example, the Rabe et al. [42] found that the sudden increases of the displacement at constant load on Si-DLC film were due to the chipping out of materials with the help of the SEM video. Rzepiejewska-Malyska et al. [43] studied the deformation mechanisms of TiN, CrN, and multilayer TiN/CrN thin films on silicon substrate. The TiN thin film showed short radial cracks, whereas CrN deformed through pileup and densification of the material. For TiN/CrN, multilayer pileup and cracks were found. Heiroth et al. [44] compared the deformation mechanism of amorphous yttria-stabilized zirconia films with crystalline Y_2_O_3_ films under nanoindentation and found that the amorphous films deform plastically by shear bands, while the crystalline films reveal a brittle behavior and accommodate the load by the formation of hoop and surface cracks.

**Figure 3:**
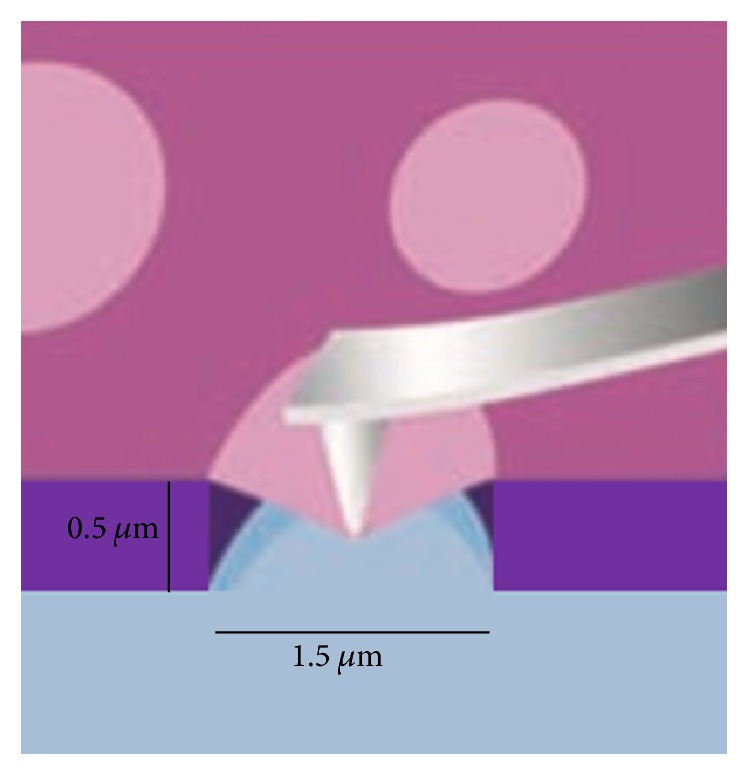
Schematic illustration of nanoindentation on freestanding graphene film [22].

As those thin films were directly deposited or grown on a substrate, the experiments yield mechanical properties of a composite structure not of the thin film itself, especially for increasingly thinner films. In order to get rid of the influence from the substrate, some researchers have conducted tests on freestanding thin films. However, the in situ SEM indentation of freestanding thin films was little reported. Lee et al. [22] measured the elastic properties and intrinsic strength of monolayer graphene with the help of AFM, as shown in Figure 3. Similar to Lee, Frank et al. [45] and Suk et al. [46] also conducted the mechanical testing on free standing thin film of graphene sheets and monolayer graphene oxide, respectively, by AFM. Leseman and Mackin [47] developed a new indentation system to investigate the freestanding Au thin films with a ball-like indentation tip. Although it was not performed inside SEM, it has the ability to record the applied load and membrane displacement simultaneously.

### 2.4. Micro/Nanoscale Bending Test

**Figure 4:**
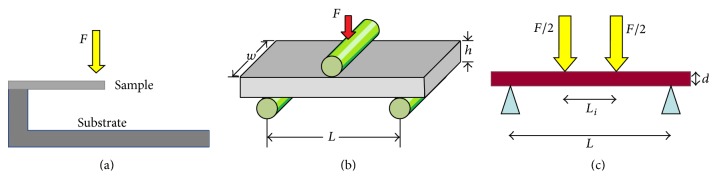
The schematic illustration of different bending tests of thin films. (a) Single point bending test. (b) Three-point double-clamped bending test and (c) four-point double-clamped bending test.

Another useful test geometry under the scope of indentation measurement is the micro/nanobending test, which can be categorized into single point bending test, three-point double-clamped bending test, and the four-point double-clamped bending test.  Figure 4 shows the typical configurations of bending test. With the help of focused ion beam (FIB) technique it is relatively easy to machine micrometer-sized bending test samples. This attracted many researchers to study the material fracture mechanism under bending test.

In single point bending test, the freestanding beam or wire is always named as cantilever. Allison et al. [48] performed in situ SEM microcantilever beam experiments on bioinspired nanocomposites and the deformation mechanism was similar to nacre. Howard et al. [49] even studied the cyclic deformation of metal microbeam under in situ SEM bending test and found that dislocation pileup within these microbeams occurs exactly as it would in a macroscopic fatigue specimen. The in situ single point bending test can also be used in the bending of nanoscale materials; for example, Vlassov et al. [50] measured Young's modulus and yield point of the Ag nanowires and even observed their plastically deformation before fracture. With the aim of providing the characterization of cracking process of metallic thin films, Hintsala et al. [51] reported the in situ doubly clamped three-point bending test of microscale and nanoscale specimens. The crack tip behavior was not kept out of view by the indenter as usual, allowing for further EBSD characterization.

What is more, with the help of newly self-developed bending test methods, some interesting phenomenon has been found by researchers. Elhebeary and Saif [52] investigated the cofabricated single crystal silicon (SCS) microbeam by a newly designed system, which eliminated any misalignment error. With the advantage of high temperature testing ability, the study revealed significant reduction in the Brittle to Ductile temperature (BDT) of SCS microbeams compared to their bulk counterparts.

## 3. In Situ SEM Electrical/Electromechanical Probing

### 3.1. In Situ Electrical Property Probing of Nanostructure

**Figure 5:**
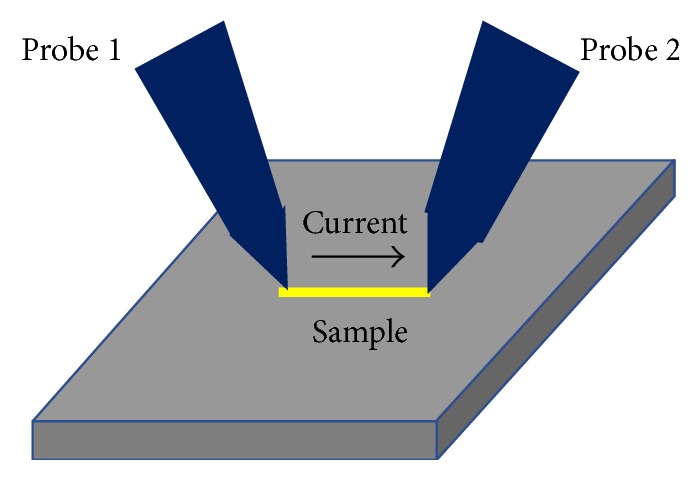
Schematic illustration of in situ electrical probing of an individual nanowire inside SEM.

Electrical property is also an important factor that affects the reliability of metallic and semiconductor nanowires beside their mechanical properties when serving as interconnecting leads and functional building blocks in applications of nanodevices and nanoelectronics [53–56]. Although it is difficult to measure the various electrical properties of nanomaterials, with the newly developed techniques, such as nanomanipulators and nanoindentation system, a lot of interesting results have been obtained, as shown in Figure 5.

Firstly, as to the fundamental *I*-*V* behaviors of nanowire, Noyong et al. [57] developed a nanomanipulation system with four manipulators and demonstrated the setup by measuring the average resistance of the platinum wire. Similar to Michael Noyong, the Au [58], GaAs [59], and CoPt/Pt [60] multilayer nanowires' resistance also have been accurately measured. Furthermore, the linear relationship between resistance and sample length [58, 60] also have been obtained which indicated that the contact resistance between tips and nanowires was largely reproducible. Another interesting phenomenon related to the current density and Joule heating of nanowires was the electromigration, which was a major reliability issue in the metallic interconnects. Huang et al. [61] studied the in situ SEM electromigration of the Cu nanowires and the relationship between the failure lifetimes and applied current densities was measured.

### 3.2. Electromechanical Coupling Analysis of Nanostructure

Electromechanical coupling effect is also a topic worth investigating in the nanomaterials. Understanding the electromechanical properties of nanomaterials is essential for further implementation of the fascinating applications in metallic and semiconducting systems. For example, increased attention has been paid to semiconducting nanowires, whose piezoresistivity [62] or piezoelectricity [63] property can be used as sensors, energy harvesting, and transistors. In electromechanical studies of nanowires, the most common approach was deforming the sample and measuring the specimen's electrical response (resistivity, generated charge, etc.) by using two or four electrical contacts same as Figure 5 shows, except that the tips of the manipulators should be bonded with the nanowires and the movement of the tips will exert tensile force and current simultaneously on the nanowire.

**Figure 6:**
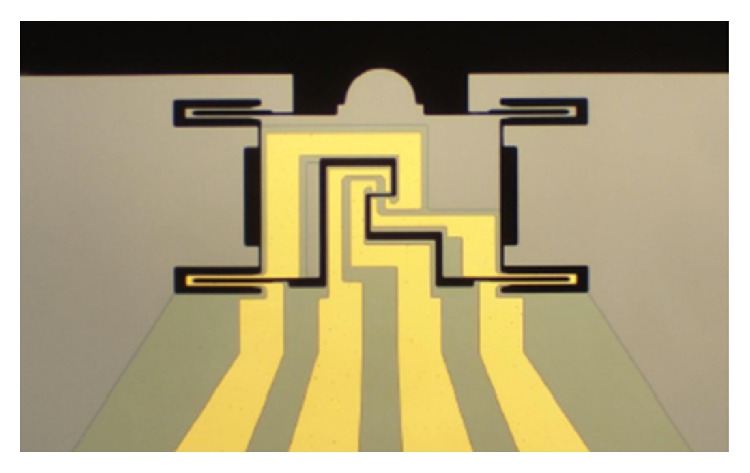
Optical image of an electrical push-to-pull micromechanical device for electromechanical coupling analysis of individual nanowires.

However, this kind of method often involves contact resistance and may introduce Schottky barriers. In order to avoid these problems, devices dedicated for electromechanical characterization have been developed. For example, by using a commercially available E-PTP (electrical push-to-pull) device with four electrodes, as Figure 6 shows, Bhowmick et al. [64] studied the ZnO nanowire under tensile stress and found that, at constant applied voltage, the current will increase with the increasing of the load force. Based on the self-designed MEMS device, Bernal et al. [65] investigated the relationship between resistance and strain of Ag and Si nanowires which have shown opposite behaviors which could be very interesting to further investigate such electromechanical coupled effect. Although the commercial devices were beneficial for speeding up the process of the measurement, self-developed devices could satisfy the different requirements of materials and structures.

## 4. The Pros and Cons Analysis of In Situ SEM Testing

Based on these above fascinating researches, the advantages of in situ SEM were very obvious. The most important one was that the comparison of the fracture process videos and the real-time data curves, such as stress-strain and *I*-*V* curves, could provide much useful information to understand the fracture mechanism of micro/nanoscale materials. It also ensured that no accidents happen, for example, debonding of the sample. Therefore, the precise and convincing results data could be guaranteed. These relatively precise results benefited with not only the advance testing platforms, but also the nanoscale resolution of the SEM images, which can provide precise measurement of the sample dimensions. Some software types, such as DIC (digital image correlation), also have lots of functions for image analysis and processing, deformation, shape, and motion measurement, which could help the researchers obtain more convincing data [66, 67].

However, the in situ SEM technique was not faultless, such as the complexity of the process and the high price of the instruments. According to our own experience, the time consumed for in situ SEM experiments was much longer than that of in situ optical ones, such as the installation of instruments in SEM, the connection between the controller outside SEM and the device through a port, and the vacuum-pumping process. Particularly, as to the sample preparation process, there are two methods to bond the materials. The first one is the FIB (focus ion beam) coating technique, which could ensure a strong and precise bonding of samples inside SEM, for example, the tensile test of Co nanowire [16]. But the manipulation of FIB is difficult to operate and the cost is high. Usually some researchers prefer to bond the materials with glue by a micromanipulator under optical microscope [18]. Although the cost is low, we need much time to practice to achieve the precise and quick bonding.

Nevertheless, for small scale samples, many tests have to be done in order to have a statistically convincing result. Few researchers paid much attention to the high-throughput problems, which were more like technique issues than scientific ones. With this aim, we have tried to speed up the process of studying the fatigue behavior of nanowires under torsion loading based on a DMD (digital micromirror device) chip, which have millions of movable micromirrors and could test many samples at one experiment [20]. As to the in situ SEM compression tests, lots of micropillars could be made on single chip at the same time, which could speed up the systematic study of the materials; for example, Moser et al. made an array of microsilicon pillars with different size [68].

## 5. Robotic In Situ SEM Micro/Nanomanipulation

As we have reviewed various in situ SEM mechanical/electrical testing progress of micro/nanomaterials above, it is worth reviewing this field from another aspect, which is about the technological manipulation components or the robotic-aid manipulation/testing instrument used in these experiments. Without the help of advanced micro/nanomanipulation instrument, it is hard to accomplish the in situ SEM characterization of nanomaterials. Owing to the precise techniques for positioning, sensing, and nanometer resolution manipulation, more and more nanorobotic manipulation systems have been installed in SEM to explore material characteristics with small scale [8, 10, 13, 18, 25, 32]. Meanwhile, a large number of researchers have engaged in developing in situ SEM nanorobotic manipulation systems for material field for several decades [45, 47, 50], since the manipulators are useful for picking, placing, bonding nanosized components, and even exerting tensile, bending, and kinking force on them. These systems can be categorized into two different types, traditional robotic manipulation systems and advanced in situ nanorobotic manipulation systems.

### 5.1. Traditional Robotic Manipulation Systems

Traditionally, the manipulation systems within in situ SEM characterization are mainly about nanoindentation system. Gane et al. have been engaged in in situ SEM material test since 1966; they developed a nanorobotic indentation system to realize in situ indentation test. In this system, the stylus is installed on a nanorobotic manipulator, which can be moved through a moving-coil device with a permanent magnet [15]. Bangert and Wagendristel have developed another kind of ultralow-load hardness tester, which is composed of elastic cantilever, electromagnet, indenter, and a double leaf spring [69]. Hedenqvist and Hogmark developed a kind of 2DOF nanorobotic manipulation system with a friction force detector and realized in situ SEM indentation test in 1997 [70]. In situ SEM tensile test helped by manipulation system first accomplished in 1999; Yu et al. developed a nanorobotic manipulation system with four degrees of freedom (DOF), which has the ability to manipulate small scale objects with one rotational DOF and three linear DOF [71]. Rzepiejewska-Malyska et al. developed a kind of nanorobotic manipulation system with three slip-stick actuators installed perpendicular to each other, which can realize in situ SEM mechanical observations during nanoindentation with high magnification [72]. Romeis et al. developed a novel nanorobotic manipulation system with two main assembly groups: an upper part which was utilized for moving the employed probe and a lower part which was composed of a force sensor and a sample support [73]. These pioneers have paved the way for fundamental material research and practical characterization.

**Figure 7:**
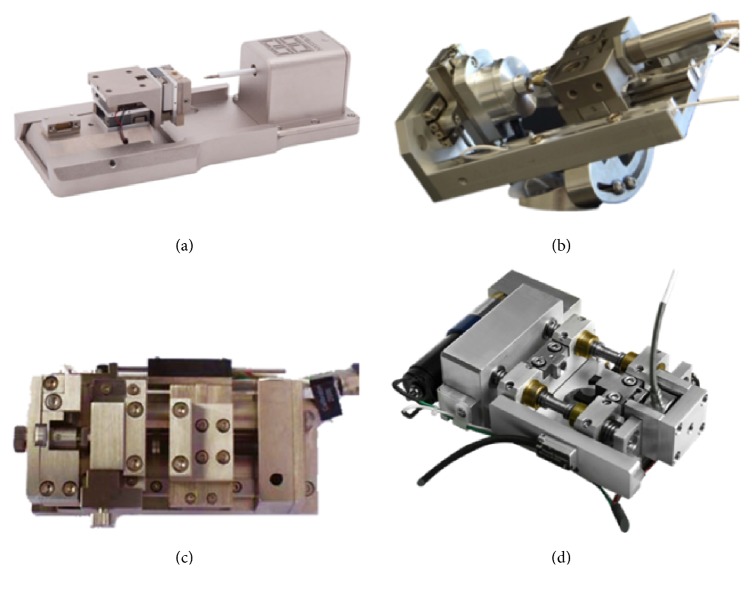
Some commercialized in situ testing systems: (a) Hysitron PI95, (b) Alemnis nanoindenter, (c) Deben Microtest, and (d) MTI Instruments tensile stage.

With the popularity of the position techniques, a number of commercial in situ SEM material characteristic test systems have been developed by companies, such as Hysitron, Alemnis, Nanomechanics, ASMEC, Kammrath & Weiss, Deben, and MTI Instruments as Figure 7 shows. With the help of these mentioned commercial in situ SEM material characteristic test systems, scientists have made a great process in material research field [15, 18, 73–75].

### 5.2. Advanced In Situ Nanorobotic Manipulation System

Compared with traditional material test nanorobotic manipulation system, scholars have developed nanorobotic manipulation platforms with multiple DOF and piezoelectric actuators to realize manipulating micro/nanoscale objects, not only for material test [76–79] but also for nanoelectromechanical systems assembly [80–82], biological cell characterization, and manipulation [83–86].

**Figure 8:**
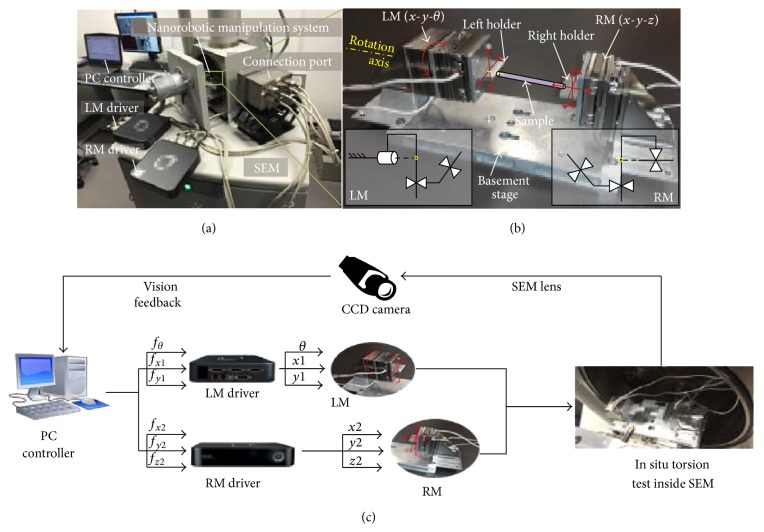
Advanced in situ SEM nanorobotics testing system developed by Shen et al. [23].

Among all kinds of advanced nanorobotic manipulation systems, actuation is one of the main challenges for scholars to control the nanorobotic manipulator precisely due to high vacuum environment inside SEM. Compared with thermal actuators, electric motors, and voice coil actuators, piezoelectric actuators are widely utilized in recent advanced nanorobotic manipulation systems because this kind of actuator does not need dissipate heat effectively and will not interfere with electron optics [87, 88]. Meanwhile, the piezoelectric actuators can generate large forces with a high bandwidth [89]. Normally, the advanced nanorobotic manipulation systems are composed of several piezoelectric actuators to realize multiple direction manipulation, as shown in Figure 8, which have both coarse positioning function and fine positioning function for working effectively [23].

Thanks to the increasingly larger chamber of model SEMs, scientists now can even combine scanning electron microscopy and atomic force microscopy (AFM) facility or nanomanipulation instruments into a single system, among which AFM/SEM hybrid systems are widely used [90]. When an AFM is integrated inside an SEM, it can realize topography analysis with high resolution and force feedback due to the real-time manipulation and imaging [91, 92]. With the help of this kind of hybrid system, manipulation and characterization of nanomaterials can be realized [10, 93–95], as well as assembly of nanodevices [96, 97] and cell characterization and manipulation [98, 99]. Owing to the advanced nanorobotic manipulation systems' development towards the direction of programmability, automation, and specificity, they will continue paving the way for micro/nanomaterial characterizations.

## 6. Summary and Outlook

This paper mainly reviewed the recent experimental efforts on in situ SEM mechanical and electrical characterization of the nanomaterials as well as the technical advances of different testing and manipulation platforms. These experiments not only manifested the unique properties of the nanomaterials but also supplied useful images or videos to help researchers in analyzing the mechanism involved, which may give beneficial guidance on their applications. Despite the significant progress, challenges still remained in the in situ SEM characterization field, such as reducing the time consumed and complexity of the experiments to produce more convincing statistical data, transferring the 2D thin films onto the testing platform effectively even with high automation, developing platforms suitable for high cycle fatigue testing, and integrating different external factors like force, electricity, and even heating into the testing platforms to study the sample's responses simultaneously. We believe further advances in both hardware and software developments will produce even smaller, delicate, more precise, and versatile testing techniques for in situ SEM characterization and make well preparation for the device applications of low-dimensional micro/nanomaterials in our daily life.

## References

[1] Chai G., Rusu E., Chow L. Microsensor on single ZnO microwire.

[2] Lee J., Kim J. (2011). Fabrication of strongly anchored, high aspect ratio elastomeric microwires for mechanical and optical applications. *Journal of Micromechanics and Microengineering*.

[3] Putnam M. C., Boettcher S. W., Kelzenberg M. D. (2010). Si microwire-array solar cells. *Energy & Environmental Science*.

[4] Yang Q., Wang W., Xu S., Wang Z. L. (2011). Enhancing light emission of ZnO microwire-based diodes by piezo-phototronic effect. *Nano Letters*.

[5] Habibi M. K., Lu Y. (2014). Crack propagation in bamboo's hierarchical cellular structure. *Scientific Reports*.

[6] Dingley D. J. (1969). A simple straining stage for the scanning electron microscope. *Micron (1969)*.

[7] Lu Y., Lou J. (2011). Quantitative in-situ nanomechanical characterization of metallic nanowires. *JOM: The Journal of The Minerals, Metals & Materials Society (TMS)*.

[8] Liu X., Liu Y., Jin B., Lu Y., Lu J. (2017). Microstructure evolution and mechanical properties of a smated mg alloy under in situ sem tensile testing. *Journal of Materials Science and Technology*.

[9] Gianola D. S., Sedlmayr A., Mnig R. (2011). In situ nanomechanical testing in focused ion beam and scanning electron microscopes. *Review of Scientific Instruments*.

[10] Zhu Y., Qin Q., Xu F. (2012). Size effects on elasticity, yielding, and fracture of silver nanowires: In situ experiments. *Physical Review B: Condensed Matter and Materials Physics*.

[11] Xu F., Qin Q., Mishra A., Gu Y., Zhu Y. (2010). Mechanical properties of ZnO nanowires under different loading modes. *Nano Research*.

[12] Zhu T., Li J., Ogata S., Yip S. (2009). Mechanics of ultra-strength materials. *MRS Bulletin*.

[13] Zhu T., Li J. (2010). Ultra-strength materials. *Progress in Materials Science*.

[14] Tian L., Cheng Y.-Q., Shan Z.-W. (2012). Approaching the ideal elastic limit of metallic glasses. *Nature Communications*.

[15] Zhang H. T., Tersoff J., Xu S. (2016). Approaching the ideal elastic strain limit in silicon nanowires. *Science Advances*.

[16] Zhang D., Breguet J.-M., Clavel R., Phillippe L., Utke I., Michler J. (2009). In situ tensile testing of individual Co nanowires inside a scanning electron microscope. *Nanotechnology*.

[17] Gu X. W., Wu Z., Zhang Y.-W., Srolovitz D. J., Greer J. R. (2013). Microstructure versus flaw: mechanisms of failure and strength in nanostructures. *Nano Letters*.

[18] Zhang H., Jiang C., Lu Y. (2017). Low-cycle fatigue testing of Ni nanowires based on a micro-mechanical device. *Experimental Mechanics*.

[19] Lu Y., Ganesan Y., Lou J. (2010). A Multi-step method for In situ mechanical characterization of 1-D nanostructures using a novel micromechanical device. *Experimental Mechanics*.

[20] Jiang C., Hu D., Lu Y. (2016). Digital micromirror device (DMD)-based high-cycle torsional fatigue testing micromachine for 1D nanomaterials. *Micromachines*.

[21] Zhang H., Siu K. W., Liao W., Wang Q., Yang Y., Lu Y. (2016). In situ mechanical characterization of CoCrCuFeNi high-entropy alloy micro/nano-pillars for their size-dependent mechanical behavior. *Materials Research Express*.

[22] Lee C., Wei X., Kysar J. W., Hone J. (2008). Measurement of the elastic properties and intrinsic strength of monolayer graphene. *Science*.

[23] Shen Y., Wan W., Zhang L., Yong L., Lu H., Ding W. (2015). Multidirectional image sensing for microscopy based on a rotatable robot. *Sensors*.

[24] Haque M. A., Saif M. T. A. (2002). Application of MEMS force sensors for in situ mechanical characterization of nano-scale thin films in SEM and TEM. *Sensors and Actuators A: Physical*.

[25] Sim G.-D., Vlassak J. J. (2014). High-temperature tensile behavior of freestanding Au thin films. *Scripta Materialia*.

[26] Zhang P., Ma L., Fan F. (2014). Fracture toughness of graphene. *Nature Communications*.

[27] Li P., Jiang C., Xu S. (2017). In situ nanomechanical characterization of multi-layer MoS. *Nanoscale*.

[28] Dowling N. E. (2012). *Mechanical Behavior of Materials*.

[29] Gane N., Bowden F. P. (1968). Microdeformation of solids. *Journal of Applied Physics*.

[30] Uchic M. D., Dimiduk D. M., Florando J. N., Nix W. D. (2004). Sample dimensions influence strength and crystal plasticity. *Science*.

[31] Greer J. R., Weinberger C. R., Cai W. (2008). Comparing the strength of f.c.c. and b.c.c. sub-micrometer pillars: Compression experiments and dislocation dynamics simulations. *Materials Science and Engineering: A Structural Materials: Properties, Microstructure and Processing*.

[32] Shan Z. W., Mishra R. K., Asif S. A. S., Warren O. L., Minor A. M. (2008). Mechanical annealing and source-limited deformation in submicrometre- diameter Nicrystals. *Nature Materials*.

[33] Greer J. R., Kim J.-Y., Burek M. J. (2009). The in-situ mechanical testing of nanoscale single-crystalline nanopillars. *JOM: The Journal of The Minerals, Metals & Materials Society (TMS)*.

[34] Dubach A., Raghavan R., Löffler J. F., Michler J., Ramamurty U. (2009). Micropillar compression studies on a bulk metallic glass in different structural states. *Scripta Materialia*.

[35] Uchic M. D., Shade P. A., Dimiduk D. M. (2009). Micro-compression testing of fcc metals: a selected overview of experiments and simulations. *JOM: The Journal of The Minerals, Metals & Materials Society (TMS)*.

[36] Kim J.-Y., Greer J. R. (2009). Tensile and compressive behavior of gold and molybdenum single crystals at the nano-scale. *Acta Materialia*.

[37] Wheeler J. M., Michler J. (2013). Elevated temperature, nano-mechanical testing in situ in the scanning electron microscope. *Review of Scientific Instruments*.

[38] Raghavan R., Wheeler J. M., Esqué-de los Ojos D. (2014). Mechanical behavior of Cu/TiN multilayers at ambient and elevated temperatures: Stress-assisted diffusion of Cu. *Materials Science and Engineering: A Structural Materials: Properties, Microstructure and Processing*.

[39] Michler J., Wasmer K., Meier S., Östlund F., Leifer K. (2007). Plastic deformation of gallium arsenide micropillars under uniaxial compression at room temperature. *Applied Physics Letters*.

[40] Oliver W. C., Pharr G. M. (2010). Nanoindentation in materials research: past, present, and future. *MRS Bulletin*.

[41] Marks L. D., Warren O. L., Minor A. M., Merkle A. R. (2008). Tribology in full view. *MRS Bulletin*.

[42] Rabe R., Breguet J. M., Schwaller P. (2004). Observation of fracture and plastic deformation during indentation and scratching inside the scanning electron microscope. *Thin Solid Films*.

[43] Rzepiejewska-Malyska K., Parlinska-Wojtan M., Wasmer K., Hejduk K., Michler J. (2009). In-situ SEM indentation studies of the deformation mechanisms in TiN, CrN and TiN/CrN. *Micron*.

[44] Heiroth S., Ghisleni R., Lippert T., Michler J., Wokaun A. (2011). Optical and mechanical properties of amorphous and crystalline yttria-stabilized zirconia thin films prepared by pulsed laser deposition. *Acta Materialia*.

[45] Frank I. (2007). Mechanical properties of suspended graphene sheets. *Journal of Vacuum Science & Technology B: Microelectronics and Nanometer Structures Processing, Measurement, and Phenomena*.

[46] Suk J. W., Piner R. D., An J., Ruoff R. S. (2010). Mechanical properties of monolayer graphene oxide. *ACS Nano*.

[47] Leseman Z. C., Mackin T. J. (2007). Indentation testing of axisymmetric freestanding nanofilms using a MEMS load cell. *Sensors and Actuators A: Physical*.

[48] Allison P., Moser R., Schirer J., Martens R., Jordon J., Chandler M. (2014). In-situ nanomechanical studies of deformation and damage mechanisms in nanocomposites monitored using scanning electron microscopy. *Materials Letters*.

[49] Howard C., Fritz R., Alfreider M., Kiener D., Hosemann P. (2017). The influence of microstructure on the cyclic deformation and damage of copper and an oxide dispersion strengthened steel studied via in-situ micro-beam bending. *Materials Science and Engineering: A Structural Materials: Properties, Microstructure and Processing*.

[50] Vlassov S., Polyakov B., Dorogin L. M. (2014). Elasticity and yield strength of pentagonal silver nanowires: In situ bending tests. *Materials Chemistry and Physics*.

[51] Hintsala E., Kiener D., Jackson J., Gerberich W. W. (2015). In-Situ Measurements of Free-Standing, Ultra-Thin Film Cracking in Bending. *Experimental Mechanics*.

[52] Elhebeary M., Saif M. T. A. Thermo-mechanical characterization of materials at micro/nanoscal E under bending.

[53] Wu Y., Xiang J., Yang C., Lu W., Lieber C. M. (2004). Single-crystal metallic nanowires and metal/semiconductor nanowire heterostructures. *Nature*.

[54] Zhou J., Gu Y., Fei P. (2008). Flexible piezotronic strain sensor. *Nano Letters*.

[55] Raskin J.-P., Colinge J.-P., Ferain I. (2010). Mobility improvement in nanowire junctionless transistors by uniaxial strain. *Applied Physics Letters*.

[56] Yan H., Choe H. S., Nam S. (2011). Programmable nanowire circuits for nanoprocessors. *Nature*.

[57] Noyong M., Blech K., Rosenberger A., Klocke V., Simon U. (2007). In situ nanomanipulation system for electrical measurements in SEM. *Measurement Science and Technology*.

[58] Peng Y., Cullis T., Inkson B. (2008). Accurate electrical testing of individual gold nanowires by in situ scanning electron microscope nanomanipulators. *Applied Physics Letters*.

[59] Fauske V. T., Kim D. C., Munshi A. M. (2014). In-situ electrical and structural characterization of individual GaAs nanowires. *Journal of Physics: Conference Series*.

[60] Peng Y., Cullis T., Luxmoore I., Inkson B. (2011). Electrical properties of individual CoPt/Pt multilayer nanowires characterized by in situ SEM nanomanipulators. *Nanotechnology*.

[61] Huang Q., Lilley C. M., Divan R. (2009). An in situ investigation of electromigration in Cu nanowires. *Nanotechnology*.

[62] He R., Yang P. (2006). Giant piezoresistance effect in silicon nanowires. *Nature Nanotechnology*.

[63] Wang Z. L., Song J. (2006). Piezoelectric nanogenerators based on zinc oxide nanowire arrays. *Science*.

[64] Bhowmick S., Stauffer D., Guo H. (2013). In situ electromechanical study of ZnO nanowires. *Microscopy and Microanalysis*.

[65] Bernal R. A., Filleter T., Connell J. G. (2014). In situ electron microscopy four-point electromechanical characterization of freestanding metallic and semiconducting nanowires. *Small*.

[66] Joo S.-H., Kato H., Jang M. J. (2017). Tensile deformation behavior and deformation twinning of an equimolar CoCrFeMnNi high-entropy alloy. *Materials Science and Engineering: A Structural Materials: Properties, Microstructure and Processing*.

[67] Kang N., Kim Y., Jeon H. (2017). Wall-thickness-dependent strength of nanotubular ZnO. *Scientific Reports*.

[68] Moser B., Wasmer K., Barbieri L., Michler J. (2007). Strength and fracture of Si micropillars: A new scanning electron microscopy-based micro-compression test. *Journal of Materials Research*.

[69] Bangert H., Wagendristel A. (1985). Ultralow-load hardness tester for use in a scanning electron microscope. *Review of Scientific Instruments*.

[70] Hedenqvist P., Hogmark S. (1997). Experiences from scratch testing of tribological PVD coatings. *Tribology International*.

[71] Yu M., Dyer M. J., Skidmore G. D. (1999). Three-dimensional manipulation of carbon nanotubes under a scanning electron microscope. *Nanotechnology*.

[72] Rzepiejewska-Malyska K. A., Buerki G., Michler J. (2008). In situ mechanical observations during nanoindentation inside a high-resolution scanning electron microscope. *Journal of Materials Research*.

[73] Romeis S., Paul J., Ziener M., Peukert W. (2012). A novel apparatus for in situ compression of submicron structures and particles in a high resolution SEM. *Review of Scientific Instruments*.

[74] Kiener D., Grosinger W., Dehm G., Pippan R. (2008). A further step towards an understanding of size-dependent crystal plasticity: in situ tension experiments of miniaturized single-crystal copper samples. *Acta Materialia*.

[75] Zhang W., Liu Y. (2012). Investigation of incremental fatigue crack growth mechanisms using in situ SEM testing. *International Journal of Fatigue*.

[76] Gong Z., Chen B. K., Liu J., Sun Y. (2014). Robotic probing of nanostructures inside scanning electron microscopy. *IEEE Transactions on Robotics*.

[77] Toh S. L., Tan P. K., Goh Y. W. (2008). In-depth electrical analysis to reveal the failure mechanisms with nanoprobing. *IEEE Transactions on Device and Materials Reliability*.

[78] Zimmermann S., Garnica Barragan S. A., Fatikow S. (2014). Nanorobotic processing of graphene: A platform tailored for rapid prototyping of graphene-based devices. *IEEE Nanotechnology Magazine*.

[79] Ru C., Zhang Y., Sun Y. (2011). Automated four-point probe measurement of nanowires inside a scanning electron microscope. *IEEE Transactions on Nanotechnology*.

[80] Dong L., Nelson B. J., Fukuda T., Arai F. (2006). Towards nanotube linear servomotors. *IEEE Transactions on Automation Science and Engineering*.

[81] Zhang Y. L., Li J., To S. (2012). Automated nanomanipulation for nanodevice construction. *Nanotechnology*.

[82] Xu D., Subramanian A., Dong L., Nelson B. J. (2009). Shaping nanoelectrodes for high-precision dielectrophoretic assembly of carbon nanotubes. *IEEE Transactions on Nanotechnology*.

[83] Shen Y., Fukuda T. (2014). State of the art: micro-nanorobotic manipulation in single cell analysis. *Rendiconti Lincei*.

[84] Shang W., Li D., Lu H., Fukuda T., Shen Y. (2017). Less-invasive non-embedded cell cutting by nanomanipulation and vibrating nanoknife. *Applied Physics Letters*.

[85] Shang W., Lu H., Wan W., Fukuda T., Shen Y. (2016). Vision-based nano robotic system for high-throughput non-embedded cell cutting. *Scientific Reports*.

[86] Ahmad M. R., Nakajima M., Kojima M., Kojima S., Homma M., Fukuda T. (2012). Instantaneous and quantitative single cells viability determination using dual nanoprobe inside ESEM. *IEEE Transactions on Nanotechnology*.

[87] Yong Y. K., Moheimani S. O. R., Kenton B. J., Leang K. K. (2012). Invited review article: high-speed flexure-guided nanopositioning: mechanical design and control issues. *Review of Scientific Instruments*.

[88] Denisyuk A. I., Krasavin A. V., Komissarenko F. E., Mukhin I. S. (2014). Mechanical, electrostatic, and electromagnetic manipulation of microobjects and nanoobjects in electron microscopes. *Advances in Imaging and Electron Physics*.

[89] Jasper D. (2012). Robot-based automation on the nanoscale. *Encyclopedia of Nanotechnology*.

[90] Shi C. (2016). Recent advances in nanorobotic manipulation inside scanning electron microscopes. *Microsystems Nanoengineering*.

[91] Russell P., Batchelor D., Thornton J. (2001). SEM and AFM: complementary techniques for high resolution surface investigations. *Veeco Metrology Group*.

[92] Häßler-Grohne W., Hüser D., Johnsen K.-P., Frase C. G., Bosse H. (2011). Current limitations of SEM and AFM metrology for the characterization of 3D nanostructures. *Measurement Science and Technology*.

[93] Fukuda T., Arai F., Dong L. (2003). Assembly of nanodevices with carbon nanotubes through nanorobotic manipulations. *Proceedings of the IEEE*.

[94] Mikczinski M. R., Josefsson G., Chinga-Carrasco G., Gamstedt E. K., Fatikow S. (2014). Nanorobotic testing to assess the stiffness properties of nanopaper. *IEEE Transactions on Robotics*.

[95] Zimmermann S., Eichhorn V., Fatikow S. Nanorobotic transfer and characterization of graphene flakes.

[96] Yang Z., Wang P., Shen Y. (2015). Dual-MWCNT probe thermal sensor assembly and evaluation based on nanorobotic manipulation inside a field-emission-scanning electron microscope. *International Journal of Advanced Robotic Systems*.

[97] Liu P., Kantola K., Fukuda T., Arai F. (2009). Nanoassembly of nanostructures by cutting, bending and soldering of carbon nanotubes with electron beam. *Journal of Nanoscience and Nanotechnology*.

[98] Ahmad M. R., Nakajima M., Kojima S., Homma M., Fukuda T. (2008). The effects of cell sizes, environmental conditions, and growth phases on the strength of individual W303 yeast cells inside ESEM. *IEEE Transactions on NanoBioscience*.

[99] Ahmad M. R., Nakajima M., Kojima S., Homma M., Fukuda T. (2011). Buckling nanoneedle for characterizing single cells mechanics inside environmental SEM. *IEEE Transactions on Nanotechnology*.

